# A Case of Trance

**Published:** 1887-12

**Authors:** 


### A CASE OF TRANCE.

A peculiar case of what is supposed to be suspended animation, has developed near Mankato. A short time ago, Miss Rosa Pfeister, seventeen years old, who has been residing for the past fifteen months with a German family two miles north of the city, retired for the night in her usual good health and buoyant spirits. The next morning, not responding to repeated calls, she was found apparently lifeless in her bed. She was still warm, and her face exactly resembled that of a sleeping person. The coroner was summoned, and, after investigating the circumstances of the supposed death, decided that no official inquiry was necessary. There was no suspicion of foul play, as the family with which the young lady resided is highly respected, though Miss Pfeister was an orphan, and had some property coming to her.

The funeral was to have occurred on a Saturday, and every preparation was made for it. When the time came, however, it was found that the remains had not begun to decompose, though they were kept in a heated room and were not packed in ice. The face had a wonderfully lifelike appearance also. After observing these and similar things, it was decided to postpone the funeral until something developed. At present, the body lies in an unchanged condition. The undertaker has made thorough tests, and finds that no decomposition has taken place either externally or internally, and he pronounces it the strangest case that he has ever met with in his long experience in such matters. He thinks that the young lady is dead, and the health officer concurs in his opinion. On the other hand, decomposition almost invariably begins within forty-eight hours after death, at the utmost, even when the body is kept in a cold room. This body has remained in a heated room for a week, and has not changed in the least during that time. The face resembles that of a sleeping person, and looks perfectly lifelike, except that it is quite pale. The house where the remains lie is crowded every day by curious spectators. The family of which the young lady was a member, is agitated by the most painful uncertainty, not knowing whether she is dead or alive. If it should prove to be a case of suspended animation, the young lady would have very narrowly escaped a horrible death, either by the knife of the post-mortem examiner or by being buried alive.—*St. Paul Pioneer-Press.*

